# Bouldering psychotherapy reduces depressive symptoms even when general physical activity is controlled for: A randomized controlled trial

**DOI:** 10.1016/j.heliyon.2018.e00580

**Published:** 2018-03-23

**Authors:** Eva-Maria Stelzer, Stephanie Book, Elmar Graessel, Benjamin Hofner, Johannes Kornhuber, Katharina Luttenberger

**Affiliations:** a Friedrich-Alexander-Universität Erlangen-Nürnberg Psychiatric and Psychotherapeutic University Clinic Erlangen, Department of Medical Psychology and Medical Sociology, Schwabachanlage 6, 91054 Erlangen, Germany; b University of Arizona, Department of Psychology, 1503 E University Blvd, Tucson, AZ 85719, USA; c Friedrich-Alexander-Universität Erlangen-Nürnberg, Department of Medical Informatics, Biometry and Epidemiology, Waldstraße 6, 91054 Erlangen, Germany; d Section Biostatistics, Paul-Ehrlich-Institut, Langen, Germany

**Keywords:** Clinical psychology, Evidence-based medicine, Psychiatry, Psychology

## Abstract

**Background:**

Bouldering psychotherapy (BPT) combines psychotherapeutic elements with physical activity (PA). It might be effective for reducing symptoms of depression, but so far, no study has assessed individuals' levels of PA to control for whether positive effects on depression can also be found when adjusting for participants' levels of PA. This is important because PA itself has been proven effective in reducing depression and therefore might be an important variable to account for – especially in therapies using sport as one therapeutic mechanism.

**Methods:**

Using a waitlist control group design, outpatients with depression were assessed at baseline and after eight, 16, and 24 weeks. The intervention group took part in an eight-week bouldering psychotherapy which met once a week for three hours. Self-report measures before and after the intervention included the Symptom Checklist-90-R (SCL-90-R), the Beck Depression Inventory (BDI-II), and the questionnaire on resources and self-management skills (FERUS). PA was assessed during the first 16-week period via FitBit Zip accelerometers.

**Results:**

Altogether, 47 complete cases (20 men and 27 women) were included in the final analyses. Depression scores dropped by up to 6.74 (CI 2.80–10.67) points on the SCL-90-R depression scale and by up to 8.26 (CI 4.21–12.31) points on the BDI-II during the BPT intervention, the control group remained stable (SCL-90-R Cohen's *d* = 0.60; BDI-II: Cohen's *d* = .50). All Participants accrued an average of 6,515 steps per day, which is considered “low-active.” Participants of the BPT intervention were significantly more likely to reduce their depressive symptoms (*p* = .025) than participants of the control group, even when PA was controlled for in a regression analysis.

**Limitations:**

Limitations of the study are the relatively small number of patients and the assessment of outcome scores via self-report.

**Conclusions:**

This study provides evidence that short-term BPT can be effective for reducing symptoms of depression even if controlled for other therapeutically active confounders including antidepressant medication, psychotherapy and general level of PA.

## 1 Background

With approximately one in eight people suffering from depression at some point in their lives (Bromet et al., 2011), depression is a very common psychiatric disorder and one of the leading causes of disability (GBD 2015 DALYs and HALE Collaborators, 2016a). It is rated one of the three leading causes of years lived with disease (YLD) for adults up to 50 years worldwide (GBD 2015 DALYs and HALE Collaborators, 2016b) and is expected to become the second leading contributor to the global burden of disease by 2020 (WHO, 2001). Given this urgency to provide depressed patients with effective and long-lasting treatment strategies, alternative therapies (e.g., exercise) that can complement traditional treatments are on the rise. Evidence-based exercise programs associated with reduced depressive scores include walking or running (Blumenthal et al., 2007; Dunn et al., 2005; Knubben et al., 2007), cycling (Dunn et al., 2005; Pinchasov et al., 2000), muscle strengthening (Mather et al., 2002; Pilu et al., 2007), stretching (Mather et al., 2002), as well as the combination of aerobic and anaerobic exercise (Brenes et al., 2007). Existing reviews and meta-analyses have verified this positive relationship between physical activity (PA) and the alleviation of depression with effect sizes ranging from 0.40 to 1.42 (Josefsson et al., 2014; Krogh et al., 2011; Rebar et al., 2015; Rimer et al., 2012; Silveira et al., 2013; Stathopoulou et al., 2006; Wegner et al., 2014). The majority of research found exercise to be comparable to the antidepressant effects of psychotherapy and medication and superior to no treatment (Cooney et al., 2013; Josefsson et al., 2014; Krogh et al., 2011; Rimer et al., 2012; Silveira et al., 2013; Stathopoulou et al., 2006; Wegner et al., 2014) with positive outcomes on psychological, social, motivational, and physiological variables (Josefsson et al., 2014; Krogh et al., 2011; Rebar et al., 2015; Rimer et al., 2012; Stathopoulou et al., 2006). Although the mechanisms underlying the exercise-depression relationship are not well understood yet, scientific evidence suggests that such mechanisms might comprise physiological as well as psychological aspects. With regard to physiological factors, the literature proposes activity-induced physiological alterations that are comparable to the effects gained through the use of antidepressant medication such as changes in the hypothalamic adrenocortical system or neurogenesis (Ernst et al., 2006; Helmich et al., 2010; Lukowski, 2013; Stathopoulou et al., 2006). The proposed psychological mechanisms include, for instance, cognitive enhancement such as distraction from negative thoughts and feelings (Craft, 2005; Searle et al., 2011), regaining a sense of purpose (Searle et al., 2011), improved performance on tasks involving executive control and perception (Voelcker-Rehage et al., 2011), enhanced physical and global self-esteem, higher quality of life (both Knapen et al., 2014), and improved coping strategies (Craft, 2005; Knapen et al., 2014).

A mode of exercise only recently found effective in the treatment of depression involves bouldering – a style of climbing characterized by short and low routes of up to four to five meters. Unlike rock climbing, bouldering is performed without a rope or harness. To protect climbers from falling and serious injuries, safety mats are placed underneath them. Bouldering gyms typically offer a variety of routes of different difficulty so that both inexperienced and experienced climbers can boulder simultaneously without being underchallenged or overstrained.

The first findings from the two-year long waitlist-controlled pilot study “Klettern und Stimmung” (“climbing and mood”; KuS) suggested positive effects of a short-term bouldering psychotherapy (BPT) intervention on the severity of depression as well as dimensions associated with depression such as anxiety, self-management, and social competence (Luttenberger et al., 2015). Further evidence for the effectiveness of rock climbing as a therapeutic approach in psychosomatic rehabilitation stems from clinical case reports, field studies, and observational studies, which have shown positive outcomes with regard to symptoms of anxiety (Mehl and Wolf, 2008) and depression (Mehl and Wolf, 2008), attention deficit hyperactivity disorder (ADHD) (Wallner, 2010), cognition (Schnitzler, 2009), the socio-emotional domain such as enhanced self-confidence (Mehl and Wolf, 2008) and better social skills (Mollenhauer et al., 2011), as well as physical health benefits (Engbert and Weber, 2011; Kim and Seo, 2015; Velikonja et al., 2010). Besides psychotherapy, one of the potentially therapeutic components of a BPT intervention is the “pure” PA. In the literature an association between physical inactivity and depression is suggested (De Mello et al., 2013; Gudmundsson et al., 2015; Mammen and Faulkner, 2013; Teychenne et al., 2010; Wielopolski et al., 2015). A recent study on longitudinal associations between PA and depressive symptoms (Gudmundsson et al., 2015) found an inverse relationship between PA and depression scores. Similarly, De Mello et al. (2013) found that individuals who did not report any PA were twice as likely to suffer from symptoms of depression and anxiety than individuals who engaged in regular PA.

As BPT includes both psychotherapeutic elements and PA, two elements effective in the treatment of depression, the current study extended the study protocol of the KuS study by additionally assessing participants' levels of PA and controlling for PA when assessing the effect of the BPT on the outcome measures. General level of PA was assessed of all patients enrolled in the study via accelerometers. Accelerometers are small and affordable tracking devices that provide users with accurate and objective information on ambulatory PA such as the number of steps accumulated per day, distance traveled, or calories burned and are therefore less subjective than self-report methods such as PA diaries or questionnaires. The aim of the study is to assess, whether BPT has an effect on depression and if the effect of BPT is independent of general levels of PA, or put differently, whether BPT is still effective in reducing depression symptoms even when taking other therapeutically active variables such as general level of PA into account.

## 2 Method

### 2.1 Bouldering intervention

**Fig. 1:**
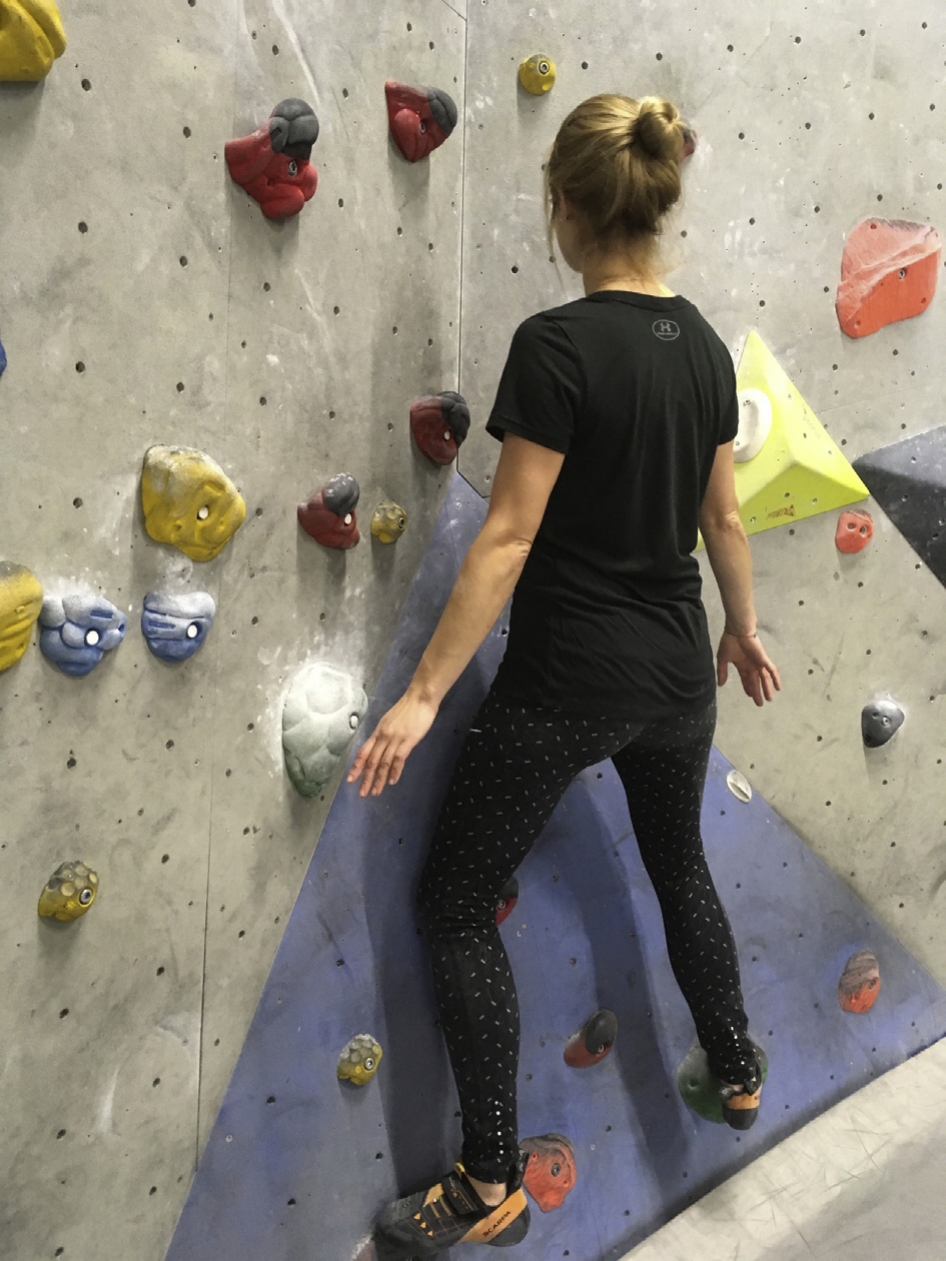
Illustration of bouldering exercise: Focus on finding the center of gravity.

The BPT intervention took place in a group format with about 12 participants in each group. Participants met once a week for eight weeks. Sessions lasted three hours from 10 a.m. to 1 p.m. with all sessions supervised by at least two mental health therapists (psychologists or registered nurses with a specific psychiatric qualification) experienced in bouldering and rock climbing. Therapists were trained in “Therapeutic rock climbing” by the Austrian “Institute for Therapeutic rock climbing” (www.therapieklettern.com). Table 1 lists the specific subjects that were covered in the eight therapeutic sessions. Each of the eight sessions focused on a specific psychological process that we believe is likely to play some role in the depression-reducing effects of BPT. Each session consisted of five parts: (1) introduction, (2) action phase 1, (3) break, (4) action phase 2, and (5) closing phase. Fig. 1 illustrates an exercise for beginners where removing the use of hand holds, while climbing, can optimize the focus on participants' center of gravity. Sessions began with a short meditation or mindfulness exercise to direct participants' attention away from their worries and to get them to focus on the current moment. For a detailed description of the treatment, please see Luttenberger et al. (2015).

**Table 1:** Overview of sessions.

Session	Topic
1	Introduction to bouldering, building group cohesion, identifying the physical abilities of participants
2	Old habits – new ways
3	Expectation versus experience, dealing with limitations
4	Self-efficacy: The power of small steps
5	Fear and trust
6	Trusting yourself and trusting others
7	Transfer to daily life
8	Reflections on lessons learned, free topic (based on the group's wishes)

### 2.2 Methods of evaluation

#### 2.2.1 Study design

The study was designed as a prospective, two-group controlled study with participants randomly assigned to the treatment group or the waitlist control group (2 × 2 design). Instead of paralleling patients to the two groups as planned initially we randomized participants following recommendations by the ethics committee. All participants wore FitBit Zip accelerometers for the first sixteen weeks (t0 – t2) to measure their levels of PA (paragraph “Measures” for more detailed information). In total, the study involved four psychological assessments that were administered at eight-week intervals. All subjects were initially assessed at baseline (t0) and after eight weeks (t1; end of the BPT for the intervention group). Then the control participants participated in the eight-week intervention while the intervention group returned to their treatment as usual (TAU), which we did not intervene with, and both groups were reassessed (t2; end of the BPT for the waitlist group). All participants were free at any time to engage in other sport activities besides the study intervention or to receive additional psychotherapy or medication. For both groups, the final assessment took place eight weeks after the waitlist group had completed the intervention (t3). This design was repeated with new participants after 16 weeks, thus resulting in a first wave (Intervention group 1 and waitlist group 1) and an additional second wave (Intervention group 2 and Waitlist group 2). This design allowed pre-post comparisons as well as group comparisons. All procedures were approved by the Friedrich-Alexander Universität of Erlangen-Nürnberg Ethics Committee (Re.-No. 99_13 B). Participation was voluntary, and subjects were free to leave the study at any time.

#### 2.2.2 Recruitment and randomization

The results reported in the following were collected from April, 2014, to February, 2015. Subjects were outpatients recruited from two psychiatric hospitals in Erlangen, locally-based psychotherapists, and other psychiatric services (i.e., support groups). At the psychiatric hospitals, informational material was posted so that either the patients themselves or their physicians could apply to participate in the study. Similarly, outpatient psychotherapists and other psychiatric services were provided with the same informational material to share with their patients. In addition, we offered information sessions that were announced via newspapers and the Internet.

**Fig. 2:**
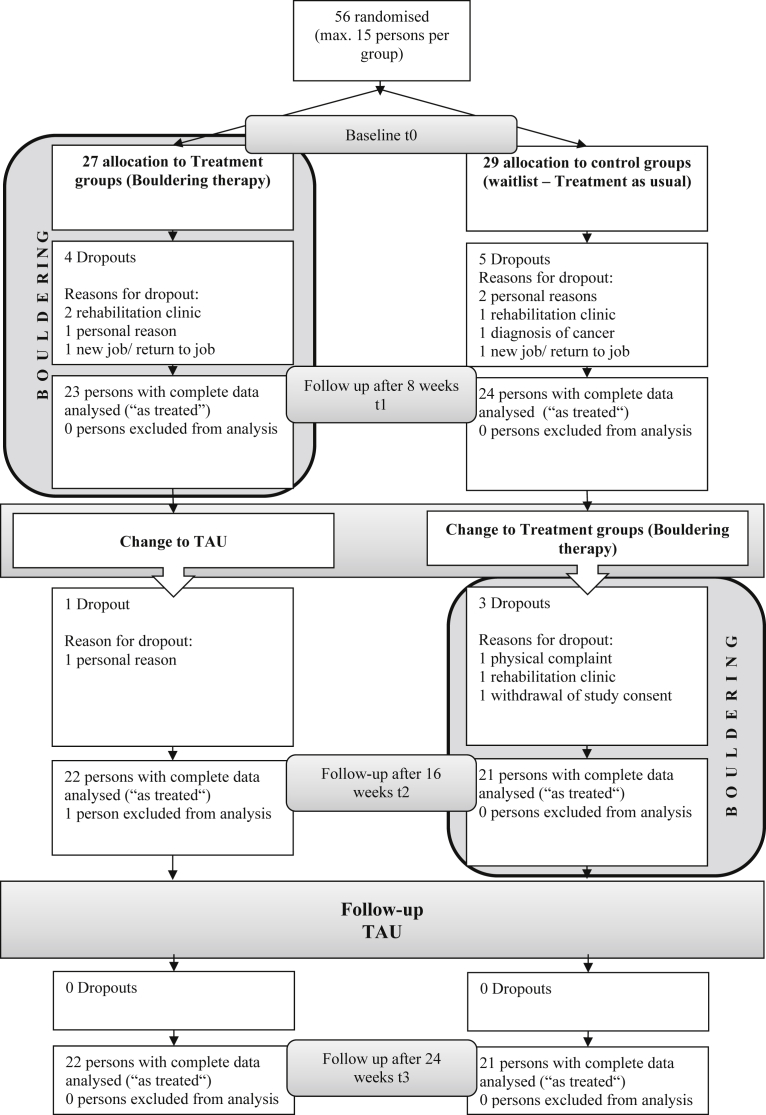
Consort flow chart.

Participants were randomly assigned to the intervention or waitlist group. We computer-generated a randomization list for each group at t0 (wave 1: Intervention group 1 and Waitlist group 1 together; wave 2: Intervention group 2 and Waitlist group 2 together, see Consort Flow Chart, Fig. 2), assigning half of the participants to the intervention group and the other half to the waitlist group. In some cases, randomization was not possible because the maximum number of participants had been reached in a group; in a few cases, if a participant was planning to miss more than two sessions in one of the two eight-week periods during the BPT intervention, he or she was assigned to the other wave.

#### 2.2.3 Inclusion and exclusion criteria

Eligibility was determined through a meeting with one of the therapists. During this meeting, we screened participants for current depressive symptoms by administering the WHO screening test on depression (WHO-5: (Bech, 2004; Henkel et al., 2003) www.who-5.org), a screening tool with very good sensitivity of around 93% to detect depression. Inclusion criteria consisted of an already existing diagnosis of depression according to the DSM-IV (APA, 2000) or a score of less than 13 points on the WHO depression scale (Bech, 2004). Those who fulfilled the inclusion criteria provided written informed consent and were randomly assigned to either the intervention or waitlist group. Exclusion criteria consisted of current hospitalization for a psychiatric disorder, acute mania, current substance abuse, endangerment of self and others, serious self-mutilation, as well as inability to exercise due to a medical condition or a Body Mass Index (BMI) of less than 18.5 or greater than 35.

#### 2.2.4 Instruments

All instruments were assessed at all measurement time points.

##### 2.2.4.1 Symptom Checklist-90-R (SCL-90-R)

The Symptom Checklist-90-R (SCL-90-R) (Franke, 2002; Schmitz et al., 2000) is a 90-item self-report inventory that measures the global intensity of psychological symptoms including depression and distress experienced during the past seven days. Nine symptom dimensions can be measured with the SCL-90-R. The 90 items are assessed on a five-point Likert scale ranging from 0 (not at all) to 4 (extremely). Ratings are summed for each subscale with higher scores indicating greater symptom severity. Scores are converted into t-scores. A t-score between 60 and 64 is slightly increased, a t-score between 65 and 69 is significantly increased, a t-score between 70 and 74 is strongly increased and a t-score between 75 and 80 is very strongly increased.

##### 2.2.4.2 The Beck Depression Inventory II (BDI-II)

The Beck Depression Inventory II (Beck et al., 1996; Hautzinger et al., 2006) is a widely used instrument designed to measure the intensity of depression in clinical and normal patients during the past two weeks. The BDI-II contains 21 specific symptoms of depression with each item listing four statements arranged by increasing severity. Responses are summed for a total score, which can range from zero to 63. A score of 13 or less indicates minimal depressive symptoms, scores from 14 to 19 represent mild depression, scores of 20–28 indicate moderate depression, and scores of 29 or above indicate severe depression.

##### 2.2.4.3 Questionnaire on resources and self-management skills (FERUS)

The FERUS is frequently used to measure individuals' health-related resources and manageability (Jack, 2007). The 66 items comprise seven scales. Subjects rate all 66 items on a five-point scale in Likert format ranging from 1 (not true at all) to 5 (extremely true). Higher test scores indicate better resources and manageability skills.

##### 2.2.4.4 Participants' data sheet

We collected baseline data on sex, age, education, BMI, experience in bouldering, as well as current treatment. Participants indicated the type of PA and the extent to which they had engaged in PA during the past eight weeks (more than once a week, once a week, once every two weeks, once a month, less than once a month) and the type and frequency of psychological and pharmaceutical treatment they had received. Further questions after the intervention period pertained to participants' satisfaction with their accelerometers (very, to some extent, not at all), their intention to continue to engage in PA after the BPT intervention, as well as unusual occurrences experienced during the past eight weeks.

##### 2.2.4.5 Assessment of physical activity other than climbing

We assessed PA by counting the number of accelerometer-based steps (Fitbit Zip) per day for 16 weeks (t0 – t2) to record participants' individual PA. Fitbit ZipTM (Fitbit Inc, USA) is a small (35.5 × 28 × 9.65 mm), light-weight (8 g), and relatively cheap accelerometer-based tracker that measures and displays steps taken, distance traveled, and calories burned. Compared with simple pedometers that display but do not record PA, accelerometers allow for broader recordings and more complex analyses of PA (e.g., intensity and duration of movement) while providing a user-friendly interface at the same time. On the Fitbit Zip, for example, a person's data accrued over the past 24 hours is shown on the tracker's display and a more detailed PA record (e.g., to view sleep patterns or to review progress over the past week) can be viewed via the company's website or mobile phone application by creating a personal account and wirelessly uploading data to the account. Prior to the baseline assessment (t0), when the participants received their Fitbit Zip, the trackers were initialized for each participant. At the baseline assessment, participants were taught how to use this device and were asked to clip the tracker to their waist or ankle or to place it in a pocket. It is important to note that participants did not receive information on how to upload their PA record to the Internet or mobile phone application. Steps were recorded from t0 until t2, thus spanning the eight weeks of the intervention and the subsequent eight weeks after the treatment for the intervention group and spanning the eight weeks prior to the BPT intervention and the eight weeks of the intervention for the waitlist group. Fitbit Zip saves data for 28 days, so every four weeks, the participants were asked to come to the study site to upload the steps accrued over the last four weeks to the study-site computer. At the end of the 16-week period, participants were asked for feedback on the Fitbit Zip and to return their accelerometers.

#### 2.2.5 Statistical analysis

We calculated descriptive statistics (frequencies, percentages, means, and standard deviations) to examine the baseline characteristics. Differences in baseline characteristics between the two groups (intervention versus waitlist) were evaluated via *χ*^2^-tests, two-sample *t*-tests, and *U*-tests.

Participants' steps per day as measured by the Fitbit Zip were recorded. At least seven days of valid data per time period (t0 – t1; t1 – t2) were required for a participant's data to be included in the analysis. A valid day was defined as a record of at least 100 steps per day. When valid data were available, we computed the median per week and consequently the mean for both time periods (t0 – t1; t1 – t2). Missing data were imputed through an EM algorithm. Changes in steps accrued between period two (t1 – t2) and period one (t0 – t1) within both groups were assessed with repeated measure *t*-tests and the nonparametric *Wilcoxon* rank sum tests. Differences between the intervention and waitlist groups were analyzed with two-sample *t*-tests and *U*-tests. Secondary outcomes as well as Fitbit-based changes in PA were viewed as exploratory. The two intervention groups (wave 1 and wave 2) and the two waitlist groups (wave 1 and wave 2) were combined for data analyses.

**Fig. 3:**
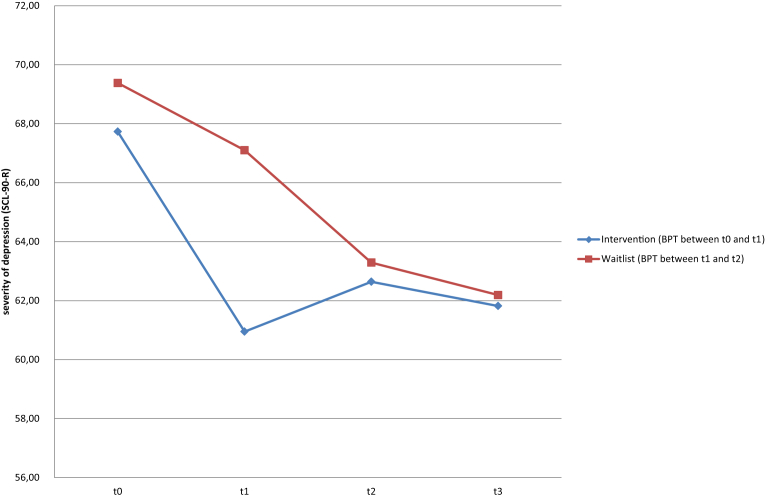
Change in SCL-90-R subscale depression scores.

Dropout analyses were computed to check for differences between the participants who dropped out and those who completed the study, using *χ*^2^-tests, two-sample *t*-tests, and *U*-tests. All analyses that included only the data from the t0 and t1 timepoints were computed with all participants who completed that time period (n = 47). All other statistical analyses were computed without the drop-outs (n = 43; Fig. 3). We checked all questionnaire items for outliers and missing values by applying frequency analyses. When fewer than 20% of a scale's items were missing, the values were replaced with the mean item score from the respective scale at that time point; this was the case for around 3% of the values across all four measurement points. When more than 20% of a scale's items were missing, the values were replaced by applying the expectation maximization (EM) algorithm (*n* = 1).

In a next step, sum scores were calculated for the SCL-90-R, FERUS, and BDI-II. First, we computed change scores as the difference between t1 and t0 and compared these scores with a two-sample *t*-test (after checking for homogeneity of variance). As a sensitivity analysis, *U*-tests were also computed. Cohen's *d* was calculated as a measure of effect size. For the main outcome criteria – depressive symptoms at t1 as measured with the SCL-90-R and the BDI-II – regression analyses were computed with sex, age, group (intervention versus waitlist), antidepressant medication, psychotherapy, severity of depression at baseline (t0), and the mean of the number of Fitbit-measured steps during the first eight-week observation period (t0 – t1) as predictors. As a sensitivity analysis, group allocation was replaced by the frequency of bouldering (control group: 0, intervention group 1–8 according to individual frequency) and additional stepwise regression analyses were conducted.

The *p*-values are considered to be exploratory. Thus, we interpreted a *p*-value of *p* ≤ 0.05 as statistically significant and also reported less stringent *p*-values of *p* ≤ 0.10. IBM SPSS Statistics 21 was used to compute all statistical analyses. Non-parametric tests were computed when assumptions (e.g., normally distributed data) for parametric tests were not met and analyses were conducted on less than 30 participants (e.g., analyses regarding changes within intervention or waitlist group), or as sensitivity analyses in addition to parametric tests.

## 3 Results

### 3.1 Description of study participants

The study period ranged from April, 2014, to February, 2015, with 56 participants fulfilling the inclusion criteria. The sample was comprised of four groups (two intervention and two waitlist groups). The waves did not differ in terms of sex (χ^2^-Test: *p* = .421), antidepressant medication (*p* = .358), or additional psychotherapy (*p* = .351) or regarding the level of depression (*t*-Test: *p* = .499 SCL depression, and .899 BDI-II) i.e. no effect of the time of testing was found. The flow of participants through the study is illustrated in Fig. 2. As displayed in this chart, the two intervention groups and the two waitlist groups were combined to increase the power of subsequent statistical procedures. 27 participants (14 female, 13 male, mean age 45.44 ± 14.12 years) were randomly assigned to the intervention group, and 29 patients (19 female, 10 male, mean age 44.41 ± 11.27 years) were assigned to the waitlist group. The average WHO well-being score was 7.16 (*SD* = 4.89), and the average BMI was 26.16 (*SD* = 5.26). Level of education was high (50% with 13 years or more of formal education). 68% of the sample indicated that they received additional psychotherapeutic treatment and/or antidepressant medication. Overall, the groups were comparable on key characteristics (Table 2). During the course of the study, 13 patients dropped out of the study for the following reasons (Fig. 2): referral to a rehabilitation clinic, personal reasons, job offer, return to employment, or cancer diagnosis. Of the 47 participants remaining after the first eight weeks, 27 (57.4%) were female, and 20 (42.6%) were male. The mean age of participants was 45 (*SD* = 12.69), and the average WHO well-being score was 7.43 (*SD* = 5.14). Two thirds (66%) of the participants underwent some form of psychotherapeutic treatment in addition to their study participation, and the same number received additional antidepressant medication. Participants who dropped out after t0 (during the first eight weeks) did not differ from the remaining sample in age, sex, BMI, additional treatment received, WHO well-being score, or PA.

**Table 2:** Sample characteristics.

Variable	Intervention group (*n* = 27)	Waitlist group (*n* = 29)	Total (*N* = 56)	Test of group differences		
				χ^2^	*T/U*	*p*
Age, *M* (*SD*)	45.44 (14.12)	44.41 (11.27)	44.91 (12.62)		0.30	.76
Sex, *n* (%)				1.08		.30
Female	14 (51.9)	19 (65.5)	33 (58.9)			
Male	13 (48.1)	10 (34.5)	23 (41.1)			
Education, *n* (%)				1.58		.90
No degree	0	1 (3.4)	1 (1.8)			
8 years	1 (3.7)	1 (3.4)	2 (3.6)			
10 years	5 (18.5)	5 (17.2)	10 (17.9)			
13 years	5 (18.5)	4 (13.8)	9 (16.1)			
University	8 (29.6)	11 (37.9)	19 (33.9)			
Vocational training	8 (29.6)	7 (24.1)	15 (26.8)			
BMI^b^, *M* (*SD*)	25.26 (4.64)	26.99 (5.73)	26.16 (5.26)		−1.24	.22
Experience bouldering, *n* (%)				2.08		.15
Yes	8 (29.6)	4 (13.8)	12 (21.4)			
No	19 (70.4)	25 (86.2)	44 (78.6)			
Additional psychotherapy, *n* (%)				0.03		.85
Yes	18 (66.7)	20 (69.0)	38 (67.9)			
No	9 (33.3)	9 (31.0)	18 (32.1)			
Antidepressants, *n* (%)				3.62		.06
Yes	15 (55.6)	23 (79.3)	38 (67.9)			
No	12 (44.4)	6 (20.7)	18 (32.1)			
WHO well-being scale^a^*M* (*SD*)	7.44 (5.43)	6.90 (4.42)	7.16 (4.89)		380.50	.86
Regular physical activity, *n* (%)				1.82		.18
Yes	22 (81.5)	19 (65.5)	41 (73.2)			
No	5 (18.5)	10 (34.5)	15 (26.8)			

a Deviated from a normal distribution (Kolmogorov-Smirnov test).

b BMI: Body Mass Index.

Of the 23 participants remaining in the intervention group, 12 (52%) individuals attended all sessions, 9 (43%) attended 5 to 7 sessions, and one person attended only 1 session.

### 3.2 Main outcome

#### 3.2.1 Primary analysis: univariate results

In the intervention group, depression scores dropped by 6.74 points on the SCL-90-R depression scale (Fig. 3) and by 8.26 points on the BDI-II during the intervention period (n = 23), whereas the waitlist group (n = 24) improved by only 1.75 points on the SCL-90-R depression scale and 2.92 points on the BDI-II during the same time period (SCL-90-R: 4.99 points difference, *t*-test: *p* = .045; BDI-II: 5.34 points difference, *t*-test: *p* = .094). The effect size was moderate with a Cohen's *d* of 0.60 for the SCL-90-R and 0.50 for the BDI-II (Table 3).

**Table 3:** Group differences between the intervention and waitlist groups for t1 – t0 for selected scales.

Scale	Intervention group (*n* = 23)	Waitlist group (*n* = 24)	Cohen's *d*	*Independent t*-test	*U*-test
	Δ*M* (*SD*)	Δ*M* (*SD*)		*p*	*p*
**Primary outcomes: Depression**					
Depression (SCL-90-R)	−6.74 (9.10)	−1.75 (7.43)	0.60	.045*	.056^†^
BDI-II Sum Score	−8.26 (9.36)	−2.92 (11.85)	0.50	.094^†^	.119
**Secondary outcomes**					
Anxiety					
Phobia/Panic: Phobic anxiety (SCL-90-R)	−5.96 (9.55)	−0.29 (8.08)	0.64	.033*	.036*
Anxiety: Anxiety (SCL-90-R)	−3.70 (8.87)	−0.08 (6.22)	0.47	.097^†^	.084^†^
Social Competence					
Interpersonal sensitivity (SCL-90-R)	−6.87 (8.84)	−1.21 (7.08)	0.71	.019*	.021*
Social support (FERUS)	3.48 (5.82)	0.96 (6.30)	−0.42	.162	.250
Self-Management					
Active and passive coping (FERUS)	7.35 (7.80)	2.38 (8.75)	−0.60	.046*	.068^†^
Self-efficacy (FERUS)	4.30 (7.31)	−0.54 (9.27)	−0.58	.053^†^	.090^†^
Self-verbalization (FERUS)	5.70 (7.97)	4.29 (7.75)	−0.18	.544	.536
**Further outcomes**					
Somatization (SCL-90-R)	−6.65 (8.24)	−0.58 (6.04)	0.84	.006*	.012*
Hostility (SCL-90-R)	−4.09 (5.63)	1.42 (9.99)	0.68	.025*	.030*
Paranoid ideation (SCL-90-R)	−5.78 (7.35)	−1.13 (5.94)	0.70	.021*	.019*
Psychoticism (SCL-90-R)	−7.78 (7.12)	−0.67 (8.52)	0.90	.003*	.005*
Hope (FERUS)	6.22 (10.41)	0.88 (7.51)	−0.59	.010*	.016*

*Note*. Differences between t0 and t1 (t1 – t0). Negative values on the BDI-II and SCL-90-R indicate improvement in symptoms; positive values on the FERUS indicate improvement in abilities. BDI-II: values between 14 and 19 indicate mild depression, and values between 20 and 28 indicate moderate depression.

^†^*p* ≤ .10. *p ≤ .05.

Improvements remained stable in the follow-up in the intervention group between t1 and t2 (SCL-90-R: *t*-test: *p* = .341; BDI-II: *t*-test: *p* = .377) as well as between t2 and t3 (SCL-90-R: *t*-test: *p* = .623; BDI-II: *t*-test: *p* = .593) and in the waitlist group between t2 and t3 (SCL-90-R: *t*-test: *p* = .526; BDI-II: *t*-test: *p* = .387).

#### 3.2.2 Sensitivity analyses: regression models

In the confounder-adjusted regression analysis (Table 4), group allocation (treatment versus waitlist) was found to have a significant effect on the change in the depression score as measured by the SCL-90-R (t1 – t0: *β* = 5.02, *p* = .025). Participants of the intervention group were more likely to improve their depressive symptoms than participants of the control group even when controlling for further, potential confounders such as PA, psychotherapy, and use of anti-depressants. Furthermore, the baseline depression score (*β* = 0.44, *p* = .002) and the estimated step count (*β* = −1.59, *p* = .004) emerged as significant predictors (the more steps, the more improvement). Receiving additional psychotherapy (individual/group) showed a trend toward significance (*β* = 3.68, *p* = .092) (utilization of psychotherapy indicating higher depression scores). Similar results were found in a regression analysis for the BDI-II where group allocation showed a relatively large effect (*β* = 6.16, *p* = 0.054) even when controlling for potential confounders, in line with the univariate analysis. Step count (*β* = −1.50, *p* = .050) showed a trend toward significance, and the baseline depression score (*β* = .46, *p* = .004) emerged as significant predictor (Table 4).

**Table 4:** Regression analysis with either the BDI-II or SCL-90-R depression scale at t1 as outcome variable.

Predictor	*β*	*Stand. b*	*p*	95% CI	
				*LL*	*UL*
**Outcome variable: Depression (SCL-90-R)**					
Sex: female	0.695	0.042	.752	−3.74	5.13
Age	0.038	0.060	.662	−0.14	0.21
Group allocation: waitlist group	−5.017	0.307	.025*	0.68	9.36
Antidepressants: no	0.213	0.012	.930	−4.70	5.13
Psychotherapy: no	3.681	0.215	.092^†^	−0.63	7.99
SCL-90-R “depression” sum score (t0)	0.442	0.440	.002*	0.18	0.70
Steps (t0 – t1)^a^	−1.585	−0.420	.004*	−2.62	−0.55
**Outcome variable: BDI**					
Sex: female	−2.755	−0.118	.376	−8.99	3.48
Age	0.182	0.206	.142	−0.06	0.43
Group allocation: waitlist group	−6.164	0.269	.054^†^	−12.44	0.11
Antidepressants: no	1.534	0.064	.672	−5.77	8.83
Psychotherapy: no	5.568	0.232	.077^†^	−0.65	11.78
BDI-II sum score (t0)	0.461	0.414	.004*	0.16	0.76
Steps (t0 – t1)^a^	−1.502	−0.284	.050*	−3.01	0.00

*Note*. CI = confidence interval; *LL* = lower limit, *UL* = upper limit; *β* = unstandardized regression coefficient; Stand. b = standardized regression coefficient. Higher scores on the outcome variables indicate more severe symptoms.

^†^*p* ≤ .10. *p ≤ .05.

a In 1,000 steps per day.

As a further sensitivity analysis instead of the group allocation, the frequency of bouldering (control group = 0, intervention group between 1 and 8) was included in a regression analysis as described above. Results showed that frequency of bouldering was highly significant (*p* = .020 with BDI and *p* = .007 with the SCL depression as dependent variable) with all other predictors remaining in the same range. The effect of frequency of bouldering (unstandardized β) was 1 point on the BDI and 0.9 points on the SCL-depression scale per bouldering session. Stepwise regression analyses support these results (data available on request).

### 3.3 Secondary outcomes

Amongst others, significant differences in improvements between the intervention and waitlist groups during the first eight weeks (t0 – t1) were found for the FERUS and SCL-90-R subscales “phobic anxiety” (SCL-90-R: *t*-test: *p* = .033, *d* = 0.64), “active and passive coping” (FERUS: *t*-test: *p* = .046, *d* = 0.60), and “interpersonal sensitivity” (SCL-90-R: *t*-test: *p* = .019, *d* = 0.71). See Table 3 for these and other selected scales.

### 3.4 Physical activity

#### 3.4.1 FitBit technology use

67.4% of the participants (n = 29) reported no technical difficulty with the tracker, 16.3% (n = 7) reported minor issues, 14% (n = 6) experienced severe technical difficulties (e.g., no display of steps, an obviously inaccurate number of steps, or low battery notifications although the battery had been changed recently), and 20.9% (n = 9) lost the tracker over the course of the 32 weeks. As described earlier, missing values were imputed by applying an EM algorithm. A sensitivity analysis revealed no meaningful differences between the original (t0 – t1: M = 7,060; t1 – t2: M = 6,858) and imputed (t0 – t1: M = 6,193; t1 – 2: M = 6,064) data sets (t0 – t1: *t*-test: *p* = .198; t1 – t2: *t*-test: *p* = .157). Overall, there was a high level of acceptability of the FitBit Zip. The majority of respondents rated the tracker as easy to use and to integrate into their daily lives.

#### 3.4.2 Steps per day

Both groups accrued around 6515 steps per day during the first measurement period (t0 – t1) with a slight increase of only 209 steps in the second measurement period (t1 – t2) for the intervention group (t0 – t1: M = 6794; t1 – t2: M = 7003; *t*-test: *p* = .558; Wilcoxon: *p* = .808) and a decrease of 553 steps in the waitlist group (t0 – t1: M = 6388; t1 – t2: M = 5835; *t*-test: *p* = .229; Wilcoxon: *p* = .033). This resulted in significant group differences in the number of accrued steps for the second measurement period (*t*-test: *p* = .03; U-test: *p* = .04) but not for the first period (*t*-test: *p* = .55; U-test: *p* = .73).

## 4 Discussion

The findings of the present study parallel and augment the outcomes of the study reported by Luttenberger et al. (2015). During the eight-week BPT intervention period, depression scores dropped by 6.74 points on the SCL-90-R and 8.26 points on the BDI-II from a highly elevated score in the clinical range to an almost normal score. Even when controlled for general PA the BPT intervention remained a significant predictor for reduced depressive symptoms.

Participants accrued 6,515 steps per day on average with levels of PA remaining fairly stable over the 16-week period. 6,515 steps per day is considered “low active” and is typical of a daily lifestyle that excludes sports/exercise according to Tudor-Locke's (Tudor-Locke and Bassett, 2004) classification.

Despite the low number of steps per day accrued by our sample, the regression analysis with the significant predictors “group” and “steps per day” (Table 4) showed that participants who engaged in a more physically active lifestyle showed lower depression scores at t1, thus indicating the importance of overall PA for mental health. Simultaneously, the regression analyses demonstrated positive effects of the BPT program applied in this study with regard to the alleviation of depressive symptoms. In interpreting these results it has to be taken into account that only allocation to BPT or waitlist was randomized. The amount of general PA and additional psychotherapy however resulted from the participants owns initiatives and was not organized by the study. Participants were free to engage in any PA besides the bouldering intervention. Hence, the significant effect on depression of general PA might either be due to its inherent antidepressant effect or due to the fact that less depressed participants were more active as a consequence. A comparable explanation applies to the negative trend of psychotherapy, where possibly the more heavily strained patients were more likely to attend psychotherapy.

Furthermore, the intervention group improved significantly more than the control group in a number of other scales such as “phobic anxiety”, “active and passive coping”, and “interpersonal sensitivity”. These results potentially reflect the fact that the BPT intervention applied in the present study comprised different therapeutic components and therefore also exerted its effects on cognitive, emotional, and social outcomes. One therapeutic component inherent in the present bouldering intervention involved aspects of mindfulness. Not only did bouldering require participants' presence in the particular moment in order to master bouldering problems but mindfulness was further fostered through short mindfulness exercises at the beginning and end of the climbing sessions. Literature offers support for the effectiveness of mindfulness in the treatment of depressive disorders (Hofmann et al., 2010). Simultaneously, bouldering may act as an exposition to subjectively threatening situations, demanding focused attention and mindfulness, and its mastery appears to be associated with feelings of self-efficacy and internal locus of control.

The effect sizes (Cohen's *d*) reported in our study were moderate and thus comparable to other short-term group therapies (McDermut et al., 2001) and to effect sizes of 0.40–1.42 found in meta-analyses on the effects of exercise on depression (Josefsson et al., 2014; Krogh et al., 2011; Rebar et al., 2015; Rimer et al., 2012; Silveira et al., 2013; Stathopoulou et al., 2006; Wegner et al., 2014).

### 4.1 Strengths and limitations of the study

#### 4.1.1 Strengths

To our knowledge, the KuS study is the first systematic investigation of the relationship between BPT and symptoms of depression using a waitlist control design. Participants were randomly allocated to either the intervention group or the waitlist group when randomization was possible. Furthermore, we applied a relatively long follow-up period (eight to sixteen weeks). In the trials reported in this paper, we supplemented the study design described by Luttenberger et al. (2015) in addition to investigating the effects of the BPT by assessing participants' levels of PA via steps per day as measured by FitBit Zips. This enabled us to control for the potential benefits of PA in general on aspects of mental health.

#### 4.1.2 Limitations

We assessed data on mental health outcomes via self-report, which is known to be prone to systematic and unsystematic biases (Bortz and Döring, 2006). However, in favor of our study, we would like to point out that we applied multivariate measures instead of focusing on single-scale instruments. Furthermore, the measures that we included in our study are commonly used in the clinical literature. We also experienced several difficulties in the assessment of PA.

First, the most well-known constraint of accelerometers is their inability to detect non-ambulatory and static activities such as weight-lifting as well as their lack of sensitivity in accounting for light-intensity or sedentary activities such as stretching (Ainsworth et al., 2015). Second, we did not control whether participants clipped their accelerometer to their waist, sock or pocket, which may impact the accuracy of steps counted. While the manufacturer recommends placement on waistband, bra, pocket or belt, only waist-worn Fitbit Zip use has been validated (e.g. Case et al., 2015; Ferguson et al., 2015; Tully et al., 2014). Third, thirteen participants reported technical difficulties (e.g., no display of steps or a dead battery), and nine participants lost their tracking device. In choosing from the various techniques for handling missing data, we decided to impute missing values via the EM algorithm, which is regarded as a valid and powerful tool that does not produce biased estimates and does not complicate subsequent analyses as is often the case for simpler techniques such as overall mean imputation (Donders et al., 2006). A sensitivity analysis that was conducted in our data set revealed no meaningful differences between the original and imputed data sets, suggesting that EM produced reasonable values in the present paper.

### 4.2 Future research perspectives

Future research should develop an integrated model that can be applied to understand the potential modes of action. Therefore, qualitative data regarding the experience of participants could complement existing research. Evidence for the mechanisms underlying therapeutic climbing or bouldering would benefit decision-making with respect to future implementations of climbing or bouldering interventions such as study design, setting, content, or time frame. Furthermore, more well-designed research studies with better control groups are needed to systematically investigate the effectiveness of bouldering psychotherapies. Future studies should additionally compare the BPT intervention with other modes of exercise such as stretching or aerobic exercise combined with psychotherapeutic interventions or each of them alone. It would also be interesting to have data on potential changes in the fitness level during BPT and their possible relations with depression. When designing bouldering research studies, it is important to keep in mind that a psychotherapeutic bouldering intervention differs greatly from the usual bouldering classes offered at climbing gyms with respect to its psychotherapeutic aspects (Luttenberger et al., 2015). Psychoeducation and therapeutic relationships, for instance, have been shown to be important variables in the treatment of major depression with positive outcomes reported for treatment adherence, psychosocial functioning, clinical improvement, as well as adaptive capacities (Tursi et al., 2013; Zuroff and Blatt, 2006). In addition, the role of social interaction and peer support derived from the BPT group should be further researched.

Moreover, it would be interesting to see if we can find similar benefits for different patient groups (e.g., outpatients as well as inpatients) as well as for mental health disorders other than depression (e.g., eating disorders). Finally, scientific evidence of long-term effects is still lacking.

## 5 Conclusions

The results of this study provide support for the argument that BPT can be efficacious in the treatment of depression. Even when taking other therapeutically active variables such as psychotherapy, antidepressant medication, and general level of PA into account, BPT still emerged as a significant predictor for the reduction of depressive symptoms. Further research on therapeutic climbing or bouldering with larger samples and controlled designs is needed to provide a greater understanding of its effectiveness. The underlying mechanisms of BPT programs have yet to be identified.

## Declarations

### Author contribution statement

Eva-Maria Stelzer: Performed the experiments; Analyzed and interpreted the data; Wrote the paper.

Stephanie Book: Performed the experiments; Wrote the paper.

Elmar Gräßel: Conceived and designed the experiments; Analyzed and interpreted the data.

Benjamin Hofner: Analyzed and interpreted the data.

Johannes Kornhuber: Conceived and designed the experiments.

Katharina Luttenberger: Conceived and designed the experiments; Analyzed and interpreted the data; Wrote the paper.

### Funding statement

This work was supported by the Psychiatric University Hospital Erlangen. We acknowledge support by Deutsche Forschungsgemeinschaft and Friedrich-Alexander-Universität Erlangen-Nürnberg within the funding programme Open Access Publishing.

### Competing interest statement

The authors declare no conflict of interest.

### Additional information

The clinical trial described in this paper was registered at ISRCTN Registry under the registration number ISRCTN17623318.

## References

[bib1] Ainsworth B., Cahalin L., Buman M., Ross R. (2015). The current state of physical activity assessment tools. Prog. Cardiovasc. Dis..

[bib2] APA (2000). Diagnostic and Statistical Manual of Mental Disorders – DSM-IV-TR.

[bib3] Bech P. (2004). Measuring the dimensions of psychological general well-being by the WHO-5. Qual. Life Newsl..

[bib4] Beck A.T., Steer R.A., Brown G. (1996). Manual for the Beck Depression Inventory-II.

[bib5] Blumenthal J.A., Babyak M.A., Doraiswamy P.M., Watkins L., Hoffman B.M., Barbour K.A., Herman S., Craighead W.E., Brosse A.L., Waugh R., Hinderliter A., Sherwood A. (2007). Exercise and pharmacotherapy in the treatment of major depressive disorder. Psychosom. Med..

[bib6] Bortz J., Döring N. (2006). Forschungsmethoden und Evaluation: für Human- und Sozialwissenschaftler.

[bib7] Brenes G.A., Williamson J.D., Messier S.P., Rejeski W.J., Pahor M., Ip E., Penninx B.W. (2007). Treatment of minor depression in older adults: a pilot study comparing sertraline and exercise. Aging Ment. Health.

[bib8] Bromet E., Andrade L.H., Hwang I., Sampson N.A., Alonso J., de Girolamo G., de Graaf R., Demyttenaere K., Hu C., Iwata N., Karam A.N., Kaur J., Kostyuchenko S., Lepine J.P., Levinson D., Matschinger H., Mora M.E., Browne M.O., Posada-Villa J., Viana M.C., Williams D.R., Kessler R.C. (2011). Cross-national epidemiology of DSM-IV major depressive episode. BMC Med..

[bib9] Case M.A., Burwick H.A., Volpp K.G., Patel M.S. (2015). Accuracy of smartphone applications and wearable devices for tracking physical activity data. JAMA.

[bib10] Cooney G.M., Dwan K., Greig C.A., Lawlor D.A., Rimer J., Waugh F.R., McMurdo M., Mead G.E. (2013). Exercise for depression. Cochrane Database Syst. Rev..

[bib11] Craft L.L. (2005). Exercise and clinical depression: examining two psychological mechanisms. Psychol. Sport Exerc..

[bib12] De Mello M.T., Lemos Vde A., Antunes H.K., Bittencourt L., Santos-Silva R., Tufik S. (2013). Relationship between physical activity and depression and anxiety symptoms: a population study. J. Affect. Disord..

[bib13] Donders A.R.T., van der Heijden G.J.M.G., Stijnen T., Moons K.G.M. (2006). Review: a gentle introduction to imputation of missing values. J. Clin. Epidemiol..

[bib14] Dunn A.L., Trivedi M.H., Kampert J.B., Clark C.G., Chambliss H.O. (2005). Exercise treatment for depression: efficacy and dose response. Am. J. Prev. Med..

[bib15] Engbert K., Weber M. (2011). The effects of therapeutic climbing in patients with chronic low back pain: a randomized controlled study. Spine J..

[bib16] Ernst C., Olson A.K., Pinel J.P., Lam R.W., Christie B.R. (2006). Antidepressant effects of exercise: evidence for an adult-neurogenesis hypothesis?. J. Psychiatry Neurosci..

[bib17] Ferguson T., Rowlands A.V., Olds T., Maher C. (2015). The validity of consumer-level, activity monitors in healthy adults worn in free-living conditions: a cross-sectional study. Int. J. Behav. Nutr. Phys. Act.

[bib18] Franke G.H. (2002). SCL-90-R. Die Symptom-Checkliste von L. R. Derogatis.

[bib19] GBD 2015 DALYs and HALE Collaborators (2016). Global, regional, and national disability-adjusted life-years (DALYs) for 315 diseases and injuries and healthy life expectancy (HALE), 1990–2015: a systematic analysis for the Global Burden of Disease Study 2015. Lancet.

[bib20] GBD 2015 DALYs and HALE Collaborators (2016). Global, regional, and national incidence, prevalence, and years lived with disability for 310 diseases and injuries, 1990–2015: a systematic analysis for the Global Burden of Disease Study 2015. Lancet.

[bib21] Gudmundsson P., Lindwall M., Gustafson D.R., Ostling S., Hallstrom T., Waern M., Skoog I. (2015). Longitudinal associations between physical activity and depression scores in Swedish women followed 32 years. Acta Psychiatr. Scand..

[bib22] Hautzinger M., Keller F., Kühner C. (2006). Beck Depressions-Inventar (BDI-II). Revision.

[bib23] Helmich I., Latini A., Sigwalt A., Carta M.G., Machado S., Velasques B., Ribeiro P., Budde H. (2010). Neurobiological alterations induced by exercise and their impact on depressive disorders. Clin. Pract. Epidemiol. Ment. Health.

[bib24] Henkel V., Mergl R., Kohnen R., Maier W., Moller H.J., Hegerl U. (2003). Identifying depression in primary care: a comparison of different methods in a prospective cohort study. BMJ.

[bib25] Hofmann S.G., Sawyer A.T., Witt A.A., Oh D. (2010). The effect of mindfulness-based therapy on anxiety and depression: a meta-analytic review. J. Consult. Clin. Psychol..

[bib26] Jack M. (2007). Fragebogen zur Erfassung von Ressourcen und Selbstmanagementfähigkeiten (FERUS).

[bib27] Josefsson T., Lindwall M., Archer T. (2014). Physical exercise intervention in depressive disorders: meta-analysis and systematic review. Scand. J. Med. Sci. Sports.

[bib28] Kim S.-H., Seo D.-Y. (2015). Effects of a therapeutic climbing program on muscle activation and SF-36 scores of patients with lower back pain. J. Phys. Ther. Sci..

[bib29] Knapen J., Vancampfort D., Morien Y., Marchal Y. (2014). Exercise therapy improves both mental and physical health in patients with major depression. Disabil. Rehabil..

[bib30] Knubben K., Reischies F.M., Adli M., Schlattmann P., Bauer M., Dimeo F. (2007). A randomised, controlled study on the effects of a short-term endurance training programme in patients with major depression. Br. J. Sports Med..

[bib31] Krogh J., Nordentoft M., Sterne J.A., Lawlor D.A. (2011). The effect of exercise in clinically depressed adults: systematic review and meta-analysis of randomized controlled trials. J Clin Psychiatry.

[bib32] Lukowski T. (2013). Sport und Psyche. Positive psychische Wirkung und wichtiger Therapiebaustein [Sports and Psyche: positive psychological effects and important adjunct in therapy]. DNP.

[bib33] Luttenberger K., Stelzer E.M., Forst S., Schopper M., Kornhuber J., Book S. (2015). Indoor rock climbing (bouldering) as a new treatment for depression: study design of a waitlist-controlled randomized group pilot study and the first results. BMC Psychiatry.

[bib34] Mammen G., Faulkner G. (2013). Physical activity and the prevention of depression: a systematic review of prospective studies. Am. J. Prev. Med..

[bib35] Mather A.S., Rodriguez C., Guthrie M.F., McHarg A.M., Reid I.C., McMurdo M.E. (2002). Effects of exercise on depressive symptoms in older adults with poorly responsive depressive disorder: randomised controlled trial. Br. J. Psychiatry.

[bib36] McDermut W., Miller I.W., Brown R.A. (2001). The efficacy of group psychotherapy for depression: a meta-analysis and review of empirical research. Clin. Psychol..

[bib37] Mehl K., Wolf M. (2008). Erfahrungsorientiertes Lernen in der Psychotherapie – evaluation psychophysischer Expositionen auf dem Hochseil im Rahmen eines multimethodalen stationären Behandlungskonzeptes [Experiential learning in psychotherapy. Evaluation of psychophysical exposure to a tightrope course as adjunct to inpatient psychotherapeutic treatment]. Psychotherapeut.

[bib38] Mollenhauer A., Doll N., Renz P., Luntz J. (2011). Therapeutisches Klettern in der Akutpsychiatrie [Therapeutic climbing for acute psychiatric patients]. Pflegewissenschaft.

[bib39] Pilu A., Sorba M., Hardoy M.C., Floris A.L., Mannu F., Seruis M.L., Velluti C., Carpiniello B., Salvi M., Carta M.G. (2007). Efficacy of physical activity in the adjunctive treatment of major depressive disorders: preliminary results. Clin. Pract. Epidemiol. Ment. Health.

[bib40] Pinchasov B.B., Shurgaja A.M., Grischin O.V., Putilov A.A. (2000). Mood and energy regulation in seasonal and non-seasonal depression before and after midday treatment with physical exercise or bright light. Psychiatry Res..

[bib41] Rebar A.L., Stanton R., Geard D., Short C., Duncan M.J., Vandelanotte C. (2015). A meta-meta-analysis of the effect of physical activity on depression and anxiety in non-clinical adult populations. Health Psychol. Rev..

[bib42] Rimer J., Dwan K., Lawlor D.A., Greig C.A., McMurdo M., Morley W., Mead G.E. (2012). Exercise for depression. Cochrane Database Syst. Rev..

[bib43] Schmitz N., Hartkamp N., Kruse J., Franke G.H., Reister G., Tress W. (2000). The symptom-check-list-90-R (SCL-90-R): a German validation study. Qual. Life Res..

[bib44] Schnitzler E.E. (2009). Loslassen, um weiter zu kommen – Praxisbericht: Therapeutisches Klettern in der psychosomatischen Rehabilitation [Letting go in order to move on–clinical report: therapeutic climbing in psychosomatic rehabilitation]. Rehabilitation.

[bib45] Searle A., Calnan M., Lewis G., Campbell J., Taylor A., Turner K. (2011). Patients' views of physical activity as treatment for depression: a qualitative study. Br. J. Gen. Pract..

[bib46] Silveira H., Moraes H., Oliveira N., Coutinho E.S., Laks J., Deslandes A. (2013). Physical exercise and clinically depressed patients: a systematic review and meta-analysis. Neuropsychobiology.

[bib47] Stathopoulou M.G., Powers M.B., Berry A.C., Smits J.A.J., Otto M.W. (2006). Exercise interventions for mental health: a quantitative and qualitative review. Clin. Psychol..

[bib48] Teychenne M., Ball K., Salmon J. (2010). Sedentary behavior and depression among adults: a review. Int. J. Behav. Med..

[bib49] Tudor-Locke C., Bassett D.R. (2004). How many steps/day are enough? Preliminary pedometer indices for public health. Sports Med..

[bib50] Tully M.A., McBride C., Heron L., Hunter R.F. (2014). The validation of Fibit Zip™ physical activity monitor as a measure of free-living physical activity. BMC Res. Notes.

[bib51] Tursi M.F., Baes C., Camacho F.R., Tofoli S.M., Juruena M.F. (2013). Effectiveness of psychoeducation for depression: a systematic review. Aust. N. Z. J. Psychiatry.

[bib52] Velikonja O., Curic K., Ozura A., Jazbec S.S. (2010). Influence of sports climbing and yoga on spasticity, cognitive function, mood and fatigue in patients with multiple sclerosis. Clin. Neurol. Neurosurg..

[bib53] Voelcker-Rehage C., Godde B., Staudinger U.M. (2011). Cardiovascular and coordination training differentially improve cognitive performance and neural processing in older adults. Front. Hum. Neurosci..

[bib54] Wallner S. (2010). Psychologisches Klettern: Klettern als Mittel klinisch- und gesundheitspsychologischen Handelns [Psychological Climbing. Climbing as an instrument of clinical and health psychological treatment]. Psychol. Österreich.

[bib55] Wegner M., Helmich I., Machado S., Nardi A.E., Arias-Carrion O., Budde H. (2014). Effects of exercise on anxiety and depression disorders: review of meta-analyses and neurobiological mechanisms. CNS Neurol. Disord. Drug Targets.

[bib56] WHO (2001). The World Health Report 2001-Mental Health: New Understanding, New Hope.

[bib57] Wielopolski J., Reich K., Clepce M., Fischer M., Sperling W., Kornhuber J., Thuerauf N. (2015). Physical activity and energy expenditure during depressive episodes of major depression. J. Affect. Disord..

[bib58] Zuroff D.C., Blatt S.J. (2006). The therapeutic relationship in the brief treatment of depression: contributions to clinical improvement and enhanced adaptive capacities. J. Consult. Clin. Psychol..

